# Cross-cultural adaptation and measurement properties of the Nepali version of the DASH (disability of arm, shoulder and hand) in patients with shoulder pain

**DOI:** 10.1186/s12955-019-1105-1

**Published:** 2019-03-21

**Authors:** Sudarshan KC, Saurab Sharma, Karen Ginn, Tawfiq Almadi, Hari Subedi, Darren Reed

**Affiliations:** 1 Discipline of Biomedical Sciences, Faculty of Medicine and Health, University of Sydney, Sydney, Australia; 2 Department of Physiotherapy, Kathmandu University School of Medical Sciences, Dhulikhel, Nepal; 3 Discipline of Anatomy & Histology, Faculty of Medicine and Health, University of Sydney, Sydney, Australia; 4 Center for Musculoskeletal Outcomes Research, Dunedin School of Medicine, University of Otago, Dunedin, New Zealand

**Keywords:** Shoulder pain, Musculoskeletal pain, Measurement properties, Outcome measures, Patient-reported measures, Shoulder disability, Responsiveness, Reliability, Validity

## Abstract

**Background:**

Patient-reported outcome measures (PROMs) are important tools in both clinical practice and research. However, no upper extremity PROM to assess physical disability is available in Nepali. The most commonly used and recommended questionnaire for the shoulder is the Disability of Arm, Shoulder and Hand (DASH). Therefore, the aim of the study was to translate and cross-culturally adapt the DASH into Nepali and determine its measurement properties.

**Methods:**

The translation and cultural adaptation process followed international standard procedures. The translated Nepali version of the questionnaire (DASH-NP) was completed by 156 patients with shoulder pain from three Nepali hospitals at an initial assessment and by 121 at follow-up. A Nepali version of Global Rating of Change (GROC-NP) was completed at follow-up to dichotomise improved and stable participants. Measurement properties testing included: internal consistency (Cronbach's alpha), test-retest reliability (Intraclass Correlation Coefficient, ICC), Minimal Detectable Change (MDC), construct validity - factor analysis, hypothesis testing with the Shoulder Pain and Disability Index (SPADI) (Pearson Correlation = *r*) and responsiveness - Area Under the Curve with minimal important change.

**Results:**

Significant adaptations such as changing measurement units, activities and terminology were incorporated to improve cultural relevance. Internal consistency (α = 0.92) and test-retest reliability (ICC = 0.97, 95% CI: 0.94–0.98, *p* < 0.001) were excellent. The MDC was 11 out of 100 points. There were moderate-high positive correlations with the SPADI pain and disability items (*rs* = 0.63 and 0.81, *P* < 0.001). Four factor solution was retrieved for the DASH-NP. The Area Under the Curve was 0.69 (95% CI: 0.57 - 0.81, p < 0.001) with minimal important change of 11.2/100 points.

**Conclusions:**

The Nepali translation of the DASH is comprehensible, easy to administer via self-report or interview. It is found to be a reliable, valid, and responsive measure in patients with shoulder pain in Nepal. The DASH-NP can be used to assess shoulder pain related disability in Nepal for clinical practice or research.

**Electronic supplementary material:**

The online version of this article (10.1186/s12955-019-1105-1) contains supplementary material, which is available to authorized users.

## Introduction

Shoulder pain is a common musculoskeletal complaint, with lifetime prevalence in developed nations of up to 67% [1]. It accounts for approximately 10% of physiotherapy consultations [2] and is reported to have the longest median recovery time of all musculoskeletal injuries resulting in significant personal and economic costs through loss of functional capacity and employment [3].

Patient-reported outcome measures (PROMs) form a critical part of the assessment and treatment of shoulder pain and disability. They provide reliable, valid, and responsive outcome measures that can be used to direct treatment and monitor progress, as well as primary outcome measures for research purposes. There are reported to be over 30 PROMs for the shoulder region alone [4].

The Disability of Arm, Shoulder and Hand (DASH) is one of the most commonly used and tested upper limb PROMs [4, 5]. The DASH is a reliable and valid tool for the assessment of shoulder pain and disability both in clinical practice and research [6], and is reported to have among the best psychometric properties of all upper limb PROMs [4, 5, 7]. It is the preferred PROM if a variety of shoulder conditions and social and emotional function are to be assessed [5]. The DASH was originally developed in English, and has subsequently been cross-culturally adapted and validated into more than 50 different languages (http://www.dash.iwh.on.ca/available-translations).

Nepal is a developing nation with a large proportion (> 79%) of the population living in a rural setting and relying on subsistence farming [8]. Most of the tasks involved in the non-automated daily life and the labor-intensive agricultural practices of Nepal involve repetitive and often demanding use of the upper limb. Daily tasks may include; carrying water jugs, sweeping, collecting firewood, hand ploughing, fertilizing fields, manual planting, harvesting, and threshing, storage of crops, cutting fodder for domestic animals, milking buffalos and general care of livestock. There is an increasing number of Nepalese living in urban centers and engaging in more office-based activities, but they remain in the minority. Nepal has a growing health professional sector and anecdotal evidence suggests a high proportion of patients with shoulder complaints present to clinicians from both rural and urban areas. Clinical management of shoulder conditions and the development of health provision in Nepal could be improved if a reliable, valid, and responsive shoulder PROM to assess physical disability was available in the national language, Nepali. The availability of such an instrument would also facilitate clinical research related to shoulder and upper limb dysfunction in Nepal.

Therefore, the aim of this study was to translate and cross-culturally adapt the DASH into Nepali and to determine its reliability, validity and responsiveness. With Nepal’s poor literacy rate [8] and the difficulty of follow-up due to geographic remoteness, a secondary aim was to determine the reliability of the translated Nepali DASH (DASH-NP) when administered via an interview (face-to-face or phone) compared to the conventional self-reporting method.

## Methods

The translation and cross-cultural adaptation of the DASH from the original English version Nepali was based on the internationally recognized guidelines developed by the American Association of Orthopedic Surgeons (AAOS) Outcomes Committee [9]. Approval to translate was obtained from the original developers and the translation was completed in close collaboration with them. The study was approved by the Institutional Review Committee of Kathmandu University School of Medical Sciences, Nepal.

### Participants

To be included in this study, participants had to be over 18 years of age, be able to speak fluent Nepali, and have current shoulder pain. Shoulder pain was defined as; pain over the antero-lateral, proximal aspect of the shoulder and/ or upper arm, which was aggravated by shoulder movements. Participants were also required to test positive to one of the following; Hawkins-Kennedy test, Neer’s impingement test or maximally resisted isometric manual muscle tests (abduction, external/internal rotation). Participants were excluded if they presented with; cervical spine symptoms (pain on neck movements, pain in a dermatomal pattern and/or upper limb paraesthesia), pain of systemic or bioplastic origin. Participants were recruited from the physiotherapy outpatient department of three hospitals in Nepal; a not-for-profit rural, community-based hospital, a general urban hospital and a large orthopedic referral centre. These centers were chosen to provide a representative sample of both rural and urban participants. All volunteers were given verbal information about the study and provided with a participant information statement in Nepali to read if they were able or, if they were illiterate, to have it read to them. Literate participants provided a written consent and illiterate participants’ consent was obtained verbally and signed by a witness. A target of 150 participants was chosen to meet the requirements considered adequate to test the measurement properties of a PROM [10].

### The DASH questionnaire

The DASH is a 30-item self-reported upper limb assessment scale measuring functional limitations, symptoms (pain, tingling, stiffness and weakness), and psychological factors. Each item task is scored on a five-point Likert scale with the left anchor being ‘no difficulty’ (score 1) through to the right anchor ‘unable to do’ or ‘extremely difficult’ (score 5). A minimum of 27 of the 30 items must be completed to allow a valid calculation. The score is recorded out of 100 with higher scores indicating greater disability [11].

### Translation procedures and cross-cultural adaptation

The translation process involved five steps which are described below.

#### Forward translation

Three translators independently created a forward translation of the original version of the DASH into Nepali. All three translators were born in Nepal and bilingual in Nepali and English, with Nepali as their mother tongue. The translators included a health professional, a non-medical person, and a professional translator registered with and accredited by The National Accreditation Authority for Translators and Interpreters Ltd. (NAATI, Australia). The forward translators submitted a written report highlighting difficult phrases or items used in the original version that may not be common in Nepali or posed problems in the translation process.

#### Synthesis

The three forward translations were synthesized into a single version by three investigators, the project coordinator (SKC) and two university academics (DR and SS), all bilingual in English and Nepali and having one of these as their mother tongue. Differences in the translations were discussed among this group and the translators were consulted until a consensus on instructions, response options, and all items was reached. For items where several viable options were presented a majority choice was made between the translation options.

#### Back-translation

The Nepali synthesized version of the DASH was back translated by two native English-speakers, fluent in Nepali and naive to the purpose of the study. The first back translator held a Master’s degree in Nepali literature and the second translator was a medical professional. The back translators were requested to submit a written report highlighting any challenging or unclear phrases.

#### Expert committee review

All translators, the project coordinator (SKC) and two university academics (DR, SS), comprised the expert committee to discuss discrepancies identified in the translation process. A pre-final Nepali version of the DASH (DASH-NP) was produced.

#### Pre-testing

The first five participants completing the DASH-NP were interviewed to examine the questionnaire layout, the wording of difficult phrases identified by the expert committee in the forward and back translation process and the ease of understanding and completing the questionnaire. They reported that the DASH-NP was easy to understand and fill out. They identified Item 21 referring to sexual activity as a question that could possibly be left unanswered but gave no further suggestions regarding the 30 items in then DASH-NP. Following this process, the DASH-NP was finalized and subjected to further measurement properties testing.

### Procedure

A physiotherapist was recruited as a research assistant to facilitate data collection. The research assistant and physiotherapists from the three hospitals were trained in the purpose of the study, administration of the DASH and data management procedures.

The DASH-NP and a Nepali version of the Shoulder Pain and Disability Index (SPADI-NP) were completed at an initial assessment at the outpatient clinics prior to physiotherapy treatment and again on a follow up occasion (within 3 weeks). Literate participants completed the DASH-NP and SPADI-NP as a self-reported questionnaire while illiterate participants completed the questionnaires by an interview process. In the interview, the questions were read out and the participants were asked to select the most appropriate response with no extra cueing from the interviewer. All participants unable to return to the hospital within 3 weeks due to living in remote areas completed the DASH-NP by a phone interview.

At the follow-up assessment, participants also completed a Nepali version of the Global Rating of Change (GROC-NP) questionnaire [12]. The GROC-NP uses a 7-point scale to assess the self-perceived change of the participant’s shoulder condition. The 7-point GROC is commonly used in research [13] and is the most common tool used to dichotomize individuals with shoulder pain into stable and improved groups [14, 15]. The middle marker ‘4’ indicates no change in symptoms, scores > 4 indicate progressive increments of improvement (small, moderate and large improvements) and scores < 4 worsening symptoms (small, moderate and large worsening). A change of one point is considered important change [13].

### Measurement properties analyses

Data was entered into an excel spread sheet and later transferred to SPSS version 24 for statistical analyses.

### Reliability

*Internal consistency* determines the homogeneity of the subscales within a questionnaire. The internal consistency of the DASH-NP was assessed by Cronbach’s alpha (α). Cronbach’s alpha scores of 0.50–0.69 were considered poor, 0.70–0.79 acceptable, 0.80–0.89 good and > 0.90 excellent [16].

*Test–retest reliability* was evaluated using Intraclass Correlation Coefficient (ICC_2,1_) between the initial and follow up assessment of the DASH-NP scores of participants in the stable group. Secondary analysis was done on participants who completed the initial assessment by self-administration and follow-up by interview. Scores of < 0.40 were considered poor, 0.40–0.59 fair, 0.60–0.74 good and > 0.75 excellent [17].

*Minimal detectable change* (MDC), is a measure of the variation in a scale due to measurement error. A change score can only be considered to represent a real change if it is larger than the MDC [18]. The MDC of the DASH-NP was calculated using the following formula MDC = z x √2 x SEM, where z = 1.96 (z score for estimating a 95% confidence interval), √2 represents the two DASH-NP measurements and SEM is the standard error of measurement calculated using the formula; SEM = SD (1 - ICC)^1/2^ where SD is the standard deviation for the mean change of DASH-NP score from baseline to follow-up measurement and ICC is the Intraclass Correlation Coefficient of the stable group [18].

### Validity

*The construct validity* of the DASH-NP was examined in three ways as described below.

i) Comparing the pain and disability items of the DASH-NP with the corresponding construct items in the SPADI using Pearson correlations. Additionally, the difference between DASH-NP scores at baseline and follow up assessment with the score of the GROC-NP were compared. A value < 0.35 represents low, 0.36–0.67 moderate, 0.68–0.89 high and ≥ 0.90 very high correlation [19]. The priori hypothesis was that there would be a high positive correlation between the DASH-NP and SPADI-NP items testing the same construct and a moderate negative correlation to the GROC-NP scores.
ii) Testing the mean change scores of DASH-NP *within* the improved group by using a one-sample *t*-test and then *between* groups using an independent sample *t-*test [18]. It was hypothesized that there would be a significant difference in the DASH score in the group that “improved” and secondly in the DASH scores between the stable group and the improved group at a significance level of *p* < 0.05.
iii) By factor analysis with suitability of data determined by performing correlations between the scale items [20]. The Kaiser-Meyer-Oklin sample adequacy index was tested, with a threshold value greater than 0.60 considered acceptable [21]. Bartlett’s sphericity test was performed and checked for significance [22]. Principal component analysis was conducted to examine the component structure of the 30 items of the DASH-NP questionnaire. Components with high eigenvalues (> 1.0) were extracted and all eigenvalues plotted on a Cattell’s Scree plot [23]. A break point in the data, where the curve began to level was established by visual inspection of the graph. The number of data points above the break represented the components retained for extraction and rotation. These components were then rotated by the Varimax rotation [24]. Items with loadings above 0.32 were assumed to load on a given factor [25].

### Responsiveness

Primary analysis for responsiveness was assessed by plotting Receiver Operating Characteristic (ROC) curves for the differences of DASH-NP scores at baseline and follow-up assessment between the stable (GROC 4) and improved groups (GROC 5–7). Secondary analyses were performed separately between the stable group and the small (GROC 5), medium (GROC 6) and large (GROC 7) improved groups [26]. ROC curves represent the optimal trade-off between sensitivity and specificity for detecting clinical improvement with an area under the curve (AUC) of < 0.5 indicating an instrument that does not discriminate for improvement and value of AUC = 1.0 indicating perfect discrimination between the two measures. The further the ROC curve is in the upper left corner of the graph, the higher the responsiveness of the instrument [14, 27]. The minimal important change (MIC) is the number of scores required to denote a clinically meaningful change and is extracted from the ROC curve.

## Results

### Participants

**Fig. 1:**
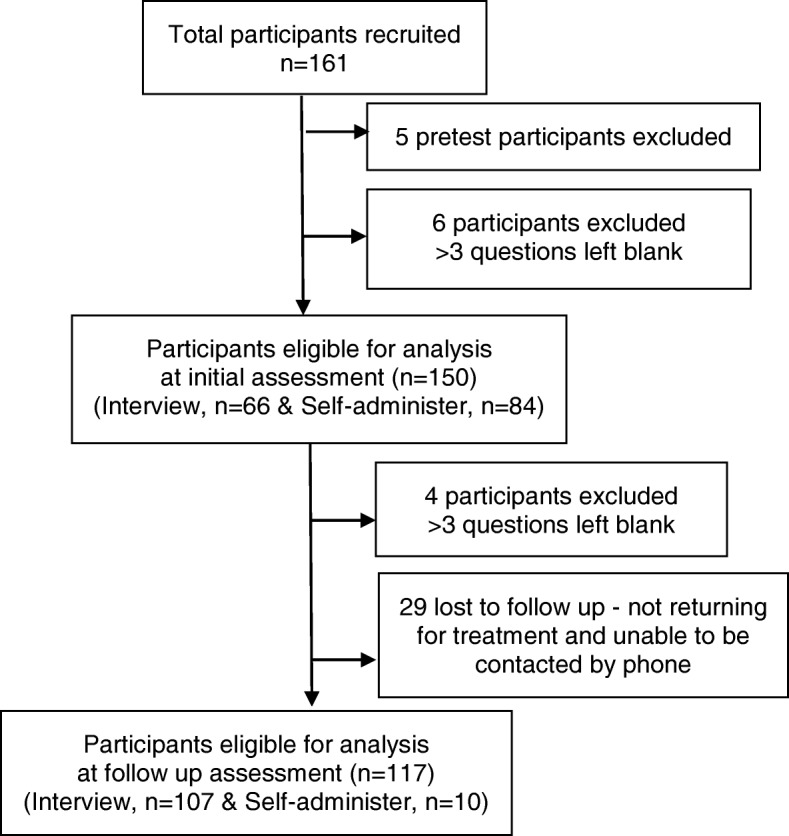
Participant flow diagram

One hundred and sixty-one participants (85F:76M, 47.7 ± 13.5 yrs) were recruited from the three physiotherapy outpatient departments (52 from the community hospital, 77 from the large specialist hospital and 32 from the general hospital). The participants were engaged in the following occupations; business owner (18%), agriculture (11%), office worker (7%), students (4%) and other - including home duties (60%). Figure 1 summarizes the number of participants completing the DASH-NP at each assessment and by self-report and interview.

Thirty-one participants were classified as stable with a GROC-NP score of ‘4’ and 86 as improved with GROC-NP scores of 5, 6 or 7. No participants reported a score below 4. Of the stable group 15 completed the questionnaires at both assessments by interview, 15 by self-report then interview and one by self-reporting at both assessments.

### Translation process and cross-cultural adaptation

Significant changes made to items in the translation and adaptation process fell into three main categories: i) measurement units converted to the metric system used in Nepal eg ‘10 lb’ to ‘5 kg’, ii) activities not common in Nepal modified to reflect local practice eg. ‘blow drying the hair’ was replaced with ‘drying hair with a towel’ as hair dryers are not used in rural areas and also uncommon in the urban areas, iii) terminology changed, eg ‘backyard’ was replaced with ‘field’ as even a small area of land at the back of house is referred to as a field and used as an area for planting crops in Nepal. After incorporating these changes, the final version of the DASH-NP was created which is available as online Additional file 1.

Three or more questions in the DASH-NP were left blank by 10 (3.5%) participants at either the initial or follow-up assessment and were excluded from further analysis. Question 18 and 19 describing recreational activities (in which you take some force or impact through your arm, shoulder or hand, e.g., batting in cricket and in which you move your arm freely, e.g., badminton) were both left blank by 10% of participants. Question 21 (Sexual activities) was left blank by 39% of all participants including 52% of those that were interviewed. Reasons given for omission included ‘uncommon activity in the rural areas’ for questions 18 and 19 and ‘culturally sensitive’ for question 21.

### Psychometric properties

#### Reliability

##### Internal consistency

The overall Cronbach’s α for DASH-NP was 0.92 (self-reporting subgroup 0.90; interview subgroup 0.94).

##### Test-retest reliability

The mean time interval between the initial and follow-up assessments was 13 ± 10 days. The ICC for DASH-NP was 0.97 (95% CI: 0.94 – 0.98, *p* < 0.001, *n* = 31) with a MDC of 11 points out of 100. Similar ICCs were recorded, when the overall group was divided into those who completed the initial assessment by self-reporting and the follow-up by interview (0.96, *n* = 15) and when both were by completed by interview (0.94, n = 15).

#### Validity

Mean change scores between the stable group and improved groups and within the improved group are shown in Table 1.

**Table 1:** Responsiveness of the DASH-NP

	N	AUC (95%CI)	MIC	Sensitivity	Specificity	Mean change score ± SD
Primary Analysis (GROC 4 vs 5–7)	31/86	0.69* (0.57–0.81)	11.2	0.55	0.85	5.1 ± 4.1
Small improvement (GROC 4 vs 5)	31/55	0.61 (0.50–0.73)	8.6	0.40	0.87	1.8 ± 4.4
Medium improvement (GROC 4 vs 6)	31/21	0.67* (0.49–0.83)	9.5	0.48	0.93	9.7 ± 5.2
Large improvement (GROC 4 vs 7)	31/10	0.97* (0.97–1.00)	14.0	0.90	0.99	15.9 ± 5.3

*Abbreviations*: *DASH-NP* Disability of Arm Shoulder and Hand, *GROC* Global Rating of Change, *AUC* Area Under the Curve, *MIC* Minimum Important Change, *SD* Standard Deviation. *Indicates significance *p* < 0.05

##### Construct validity

**Fig. 2:**
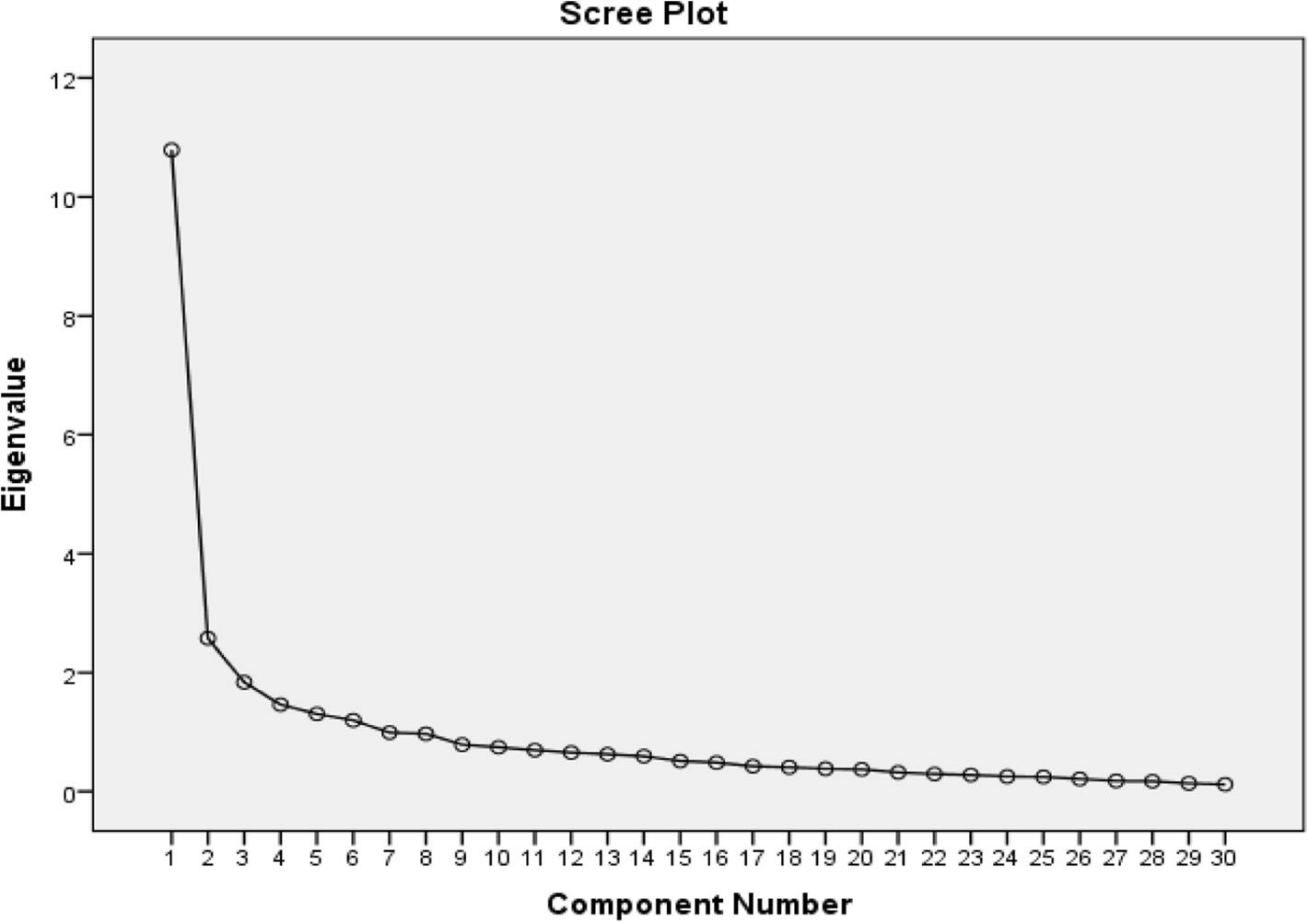
The Scree Plot of items of DASH-NP

i) There was a moderate positive correlation between the DASH and SPADI pain items *r* = 0.63, *P* < 0.001 *n* = 156 and a high positive correlation between the disability items; *r* = 0.81, P < 0.001, *n* = 132. The mean change of the DASH-NP scores demonstrated ‘moderate’ negative correlation with GROC-NP scores (*r* = − 0.40, *p* < 0.001).
ii) The single sample t-test demonstrated a significant within sample difference of mean DASH-NP scores at baseline and follow-up in the improved group [t = 7.0 (df = 85, *p* < 0.001), Mean ∆X = 10.60, 95% CI 7.60–13.9]. The independent sample t-test also revealed a significant difference between the stable and improved groups [t = 2.90 (df = 115, *p* < 0.05), Mean ∆X = 7.70, 95% CI 2.90–12.90)].
iii) *Factor Analysis -* An examination of the correlation matrix of DASH-NP items showed many items had correlation coefficients above the threshold of 0.32. The KMO sample adequacy index was > 0.88 and Bartlett’s sphericity test was statistically significant with p < 0.001 [28], indicating the data were suitable for factoral analysis. There were four components with eigenvalues > 1, explaining 35, 8, 6 and 5% of the variance. The Scree plot (Fig. 2) shows a break after the second component, resulting in two components being retained for the varimax rotation [23].

The rotated solution (Table 2) demonstrated the existence of a simple structure with the four rotated components explaining 55% of the variance. Item 21 (Sexual activities) was the only item not loaded in a category. The components contained items that could be categorised largely into:

1. Gross motor tasks (6–15, 25, 30) which explained 35% of the variance.
2. Symptoms (22, 24, 26–30) explaining 8% of the variance.
3. Fine motor tasks (1–5, 16–17, 20, 23) explaining 6% of the variance.
4. Recreational activities (18, 19) explaining 5% of the variance.

**Table 2:** Factor Analysis

	Component			
	Gross Motor	Symptoms	Fine Motor	recreational
Q1 (Open a jar)	.135	.054	***.695***	−.002
Q2 (Write)	−.004	.117	***.586***	−.108
Q3 (Turn a key)	−.177	.114	***.735***	.044
Q4 (Prepare a meal)	.166	−.023	***.743***	−.058
Q5 (Open a door)	.155	−.046	.***701***	.059
Q6 (Put an object on a high shelf)	***.781***	.057	−.032	.125
Q7 (Heavy house chores)	***.708***	−.105	.214	.049
Q8 (Make a bed)	***.720***	−.130	.243	.070
Q9 (Yard work)	***.493***	−.073	***.455***	.066
Q10 (Carry a bag)	***.430***	−.036	***.514***	−.132
Q11 (Carry a heavy object)	***.704***	−.035	.107	−.142
Q12 (Change a light bulb)	***.822***	.108	−.120	.053
Q13 (Wash hair)	***.522***	.104	***.362***	.027
Q14 (Wash back)	***.786***	−.034	.054	.091
Q15 (Put on a sweater)	***.724***	−.024	.128	−.098
Q16 (Cut food)	.123	.035	***.653***	−.111
Q17 (Activities using little effort)	.094	−.215	***.632***	.291
Q18 (Activities using some force)	.212	−.162	.106	***.783***
Q19 (Activities requiring free arm movements)	−.186	.026	.018	***.805***
Q20 (Transport activities)	.213	.074	***.411***	.103
Q21 (Sexual activities)	.000	−.209	.087	−.096
Q22 (Interference in social activities)	−.192	***.420***	***.572***	.065
Q23 (Limitation in work or ADLs)	.190	.228	***.468***	.221
Q24 (Arm pain)	.275	**.416**	.097	.141
Q25 (Pain during specific activity)	***.585***	.178	−.130	−.092
Q26 (Tingling)	−.111	**.694**	.141	−.160
Q27 (Weakness)	.050	**.699**	.186	−.071
Q28 (Stiffness)	.158	**.676**	.057	−.133
Q29 (Difficulty in sleeping)	.114	**.515**	.249	−.115
Q30 (Feeling less capable/confident)	***.341***	**.338**	−.017	.234

Extraction Method: Principal Component Analysis

Rotation Method: Oblimin with Kaiser Normalization

a. Rotation converged in 12 iterations

Note: Bold and italic texts represent factor loading in the specific factor

#### Responsiveness

**Fig. 3:**
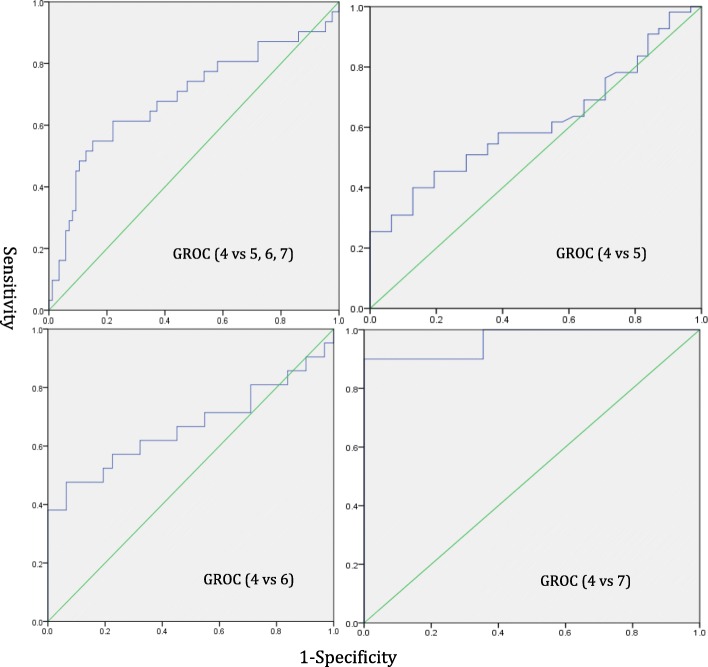
Receiver operating characteristics (ROC) curves of the stable group (GROC 4) versus the improved groups (GROC 5, 6, and 7)

Receiver operating characteristics (ROC) curves of the stable group (GROC 4) versus the improved groups (GROC 5, 6 and 7) and individually between the stable group and small (GROC 5), medium (GROC 6) and large (GROC 7) improved groups are shown in Fig. 3. The AUC and MIC with sensitivity and specificity for each of these analyses is shown in Table 1. The main analysis comparing the stable group with all improved groups demonstrated an AUC of 0.69 (95% CI: 0.57–0.81, *p* < 0.001) and MIC of 11.2 percentage points indicating that DASH-NP has acceptable responsiveness characteristics and is sensitive to detect meaningful change.

## Discussion

The DASH was successfully translated into Nepali with significant cross-culturally adaptations and the translated version was readily understood by the participants in the current study. Cross-cultural adaptions are crucial in developing a clinically meaningful and culturally relevant translation in the target language. In the current study, adaptations included changing units of measurement to metric, changing activities to ones which were similar but more commonly used in rural dominated Nepal and replacing terminology to phrases easily understood by the majority of Nepalese. Despite these adaptations up to 40% of participants left one of three questions (18, 19, 21) blank. Item 21 referring to sexual activity, a culturally sensitive topic, was left unanswered by a significant proportion of participants (39%). Interestingly a larger number of those completing the DASH-NP by interview (52%) failed to answer this question, possibly indicating an increased reluctance to reveal such information in person to others. This result is not uncommon and has been reported in other translation studies of the DASH [29] and would justify removing this question from the DASH-NP. Sporting recreation (items 18 and 19) is not a common concept among the older generation or in the rural population in Nepal and despite cultural adaptations, these items were left blank by 10% of participants. Despite these item omissions only 3.5% of individuals were excluded from data analysis due to three or more unanswered DASH items.

The DASH-NP demonstrated excellent test-retest reliability (ICC = 0.97) and internal consistency (Cronbach’s α = 0.92). These results are comparable to the values achieved on the original English version of the DASH (0.97 and 0.96 respectively) [6, 30] and in previous translations of the DASH [11, 29]. The DASH-NP was administered at the initial assessment by interview to 66 (44%) of the participants. This was deemed necessary to include the large illiterate population of Nepal which is estimated as high as 73% in some districts [8]. Cronbach’s α was computed separately for the subgroups of participants who completed the DASH-NP by self-administration and interview with excellent results for both (0.91 and 0.94 respectively), indicating that internal consistency is not affected by the method in which the DASH-NP is administered. Similarly, the ICC results when the DASH-NP was administered by self-administration and interview (0.96) or interview and interview (0.94) indicate excellent reliability of the DASH-NP regardless of the method of administration. Albeit using small sample sizes the results of this subgroup analysis indicate that the DASH-NP can be used by either self-administration or interview methods and still maintain excellent reliability.

Good construct validity of the DASH-NP was demonstrated in this study by moderate-high correlation between the corresponding pain and disability items on the DASH-NP and SPADI-NP and the overall scores of the DASH-NP with the GROC-NP. This confirmed the priori hypotheses and supports the ability of the DASH-NP to measure the constructs intended. Factor Analysis confirmed four broad categories within the DASH-NP: fine and gross motor tasks, recreational activities and symptoms. Some overlap was shown with items 9, 10, 13 referring to yard work, carrying a bag and washing hair primarily loading in the gross motor component but also in the fine motor component. This is a reasonable result considering the wide variety of tasks that could be referred to in these items and different techniques used to achieve them. Some items however, were unusually loaded including items 5 (opening a door), item 20 (transport activities) in the fine motor component and item 25 (pain during specific activity) into the gross motor component. Other studies have reported four factors for the DASH items [31, 32], but no studies have demonstrated exactly the same breakdown in the loadings, suggesting small variations are to be expected without compromising the validity of the questionnaire. Factor analysis also revealed that item 21 (sexual activity) did not load in any category and considering the tendency for this question to be left blank, it would seem reasonable to exclude this question from the Nepali DASH.

Responsiveness measured using ROC curves indicated a significant and acceptable AUC (0.69) and MIC (11.2 points) between the stable and improver groups. This is very similar to the MIC of the original English version of the DASH (10.8) [33]. More importantly, the MIC value in the current study is higher than the MDC indicating that it exceeds measurement error and gives a valid measure of clinically meaningful change over time [14, 15]. The 7- point GROC is commonly used as an outcome measure in clinical research [13] and is the most common tool used to dichotomize shoulder pain sufferers into stable and improver groups [14, 15]. Yet the ability of the GROC to differentiate small changes in symptoms has been questioned due to recall bias over time [14, 34] and its use may be viewed as a study limitation. However, the mean change scores and MIC incrementally increased with higher GROC scores and the validity evidence reported in this study suggests that the GROC was able to discriminate between the stable and improver groups. The absence of any participant recording a worsening score on the GROC-NP may indicate the inadequacy of the GROC-NP to differentiate worsening conditions perhaps due to a tendency in the Nepali culture to ‘please’ those in authority.

This study demonstrated excellent utility of the DASH-NP, with the inclusion of a wide age range (47.7 ± 13.5 yrs) of both literate and illiterate participants and equal representation of male and females (85F:76M). Occupational data was also representative of Nepal with only 29% of participants in sedentary occupations (such as office-workers, students or small business owners) while 71% were involved in agriculture or other activities such as home duties. Although not specified in data collection, in Nepal home duties often includes cooking, manually cleaning/ sweeping the house and surrounds, taking care of children and elderly, washing clothes by hand, working in a kitchen garden and in addition in more rural areas tending the fields and domestic animals (eg buffalo), collecting water and firewood.

## Conclusion

The Nepali translation of the DASH is comprehensible, easy to administer via self-report or interview. It is found to be a reliable, valid, and responsive measure in patients with shoulder pain in Nepal. The study offers evidence that the DASH-NP can be administered via an interview (face-to-face or phone) or as conventional self-reporting method without compromising its reliability. The DASH therefore can be used in both clinical practice and research to assess physical disability in patients with shoulder pain in Nepal.

## Additional file

Additional file 1: Nepali translation of DASH. (PDF 479 kb)

## Abbreviations

AAOS: American Association of Orthopedic Surgeons Outcomes Committee
AUC: Area Under the Curve
DASH: Disability of Arm, Shoulder and Hand questionnaire
DASH-NP: Nepali version of the Disability of Arm, Shoulder and Hand questionnaire
GROC-NP: Nepali version of the Global Rating of Change scale
ICC: Intraclass correlation coefficient
MDC: Minimal Detectable Change
MIC: Minimal Importance Change
PROMs: Patient-Reported Outcome Measures
ROC: Receiver Operating Characteristic Curves
SD: Standard Deviation
SEM: Standard Error of Measurement
