# Supplementary material for: Cross-cultural adaptation and measurement properties of the Nepali version of the DASH (disability of arm, shoulder and hand) in patients with shoulder pain
Source: Health Qual Life Outcomes. 2019 Mar 21;17:51. doi: 10.1186/s12955-019-1105-1 (PMC6429798; doi:10.1186/s12955-019-1105-1)
Supplement: Supplementary file 1 — Nepali translation of DASH. (PDF 479 kb) [file 12955_2019_1105_MOESM1_ESM.pdf]

पाखुरा, काँध र हातको असक्षमताहरु (DASH-NP)

ड्याश

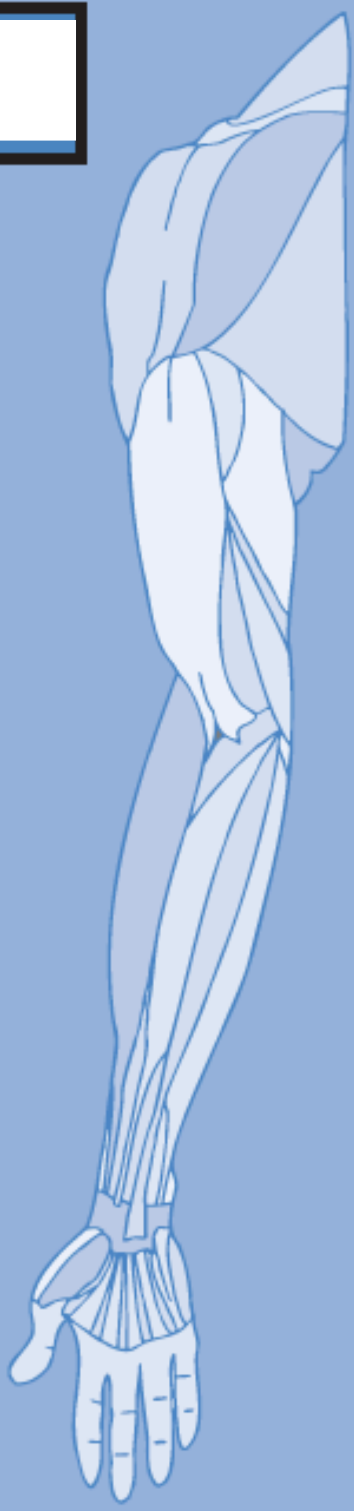

निर्देशनहरु

यो प्रश्नावलीले तपाईको लक्षणहरुको साथै केहि कार्यहरु गर्नसक्ने तपाईको क्षमताको बारेमा सोध्छ ।

गत हप्ताको तपाईको अवश्थाको आधारमा उपयुक्त संख्यामा गोलो लगाएर कृपया *प्रत्येक प्रश्नको* उत्तर दिनुहोस् ।

यदि गत हप्ता कुनै क्रियाकलाप गर्ने मौका नपाएको भए उचित जवाफमा *सबैभन्दा राम्रो अनुमान* लगाउनुहोस् ।

तपाईले जुन हात प्रयोग गरेर कार्य गरेपनि फरक पर्दैन ; तपाईले सो कार्य जसरी गरे पनि कृपया आफ्नो क्षमताको आधारमा उत्तर दिनुहोस् ।

पाखुरा, काँध र हातको असक्षमताहरु (DASH-NP)

गत हप्ता निम्न क्रियाकलापहरु गर्न सक्ने आफ्नो क्षमताको मुल्यांकन गर्न कृपया उपयुक्त जवाफ मुनि भएको अंकमा गोलो लगाउनुहोस् ।

	गाह्रो छैन	थोरै गाह्रो	मध्यम गाह्रो	धेरै गाह्रो	गर्न सक्दिँन
१. कस्सिएको अथवा नयाँ बट्टाको बिर्को खोल्न	१	२	३	४	५
२. लेख्न	१	२	३	४	५
३. साँचो घुमाउन	१	२	३	४	५
४. खाना पकाउन	१	२	३	४	५
५. धकेलेर गह्रुँगो ढोका खोल्न	१	२	३	४	५
६. टाउको भन्दा अग्लो ठाउँमा सामान राख्न	१	२	३	४	५
७. गार्हो घरेलु काम गर्न (जस्तै भित्ता सफा गर्न, भुई पुछ्न)	१	२	३	४	५
८. बारी/बगैंचाको काम गर्न	१	२	३	४	५
९. ओछ्यान मिलाउन	१	२	३	४	५
१०. किनमेलको झोला बोक्न	१	२	३	४	५
११. गह्रुंगो सामान बोक्न (५ किलो भन्दा माथि)	१	२	३	४	५
१२. टाउको भन्दा अग्लो ठाउँको चिम फेर्न	१	२	३	४	५
१३. कपाल नुहाउन/सुकाउन	१	२	३	४	५
१४. ढाड नुहाउन	१	२	३	४	५
१५. टाँख नभएको स्वीटर लगाउन	१	२	३	४	५
१६. चक्कुले खानेकुरा काट्न	१	२	३	४	५
१७. थोरै प्रयासबाट गर्न सकिने समय बिताउन गरिने क्रियाकलापहरु (जस्तै, तास खेलाई, बुनाई आदि)	१	२	३	४	५
१८. समय बिताउन गरिने क्रियाकलापहरु जसमा तपाईको काँध, पाखुरा र हातको केहि बल लाग्छ अथवा ती अंगहरुमा केहि प्रभाव पर्छ (जस्तै, क्रिकेटमा ब्याटिङ गर्ने, भलिबल, हम्मर चलाउने आदि)	१	२	३	४	५
१९. समय बिताउन गरिने क्रियाकलापहरु जसमा तपाईले आफ्नो हात खुलेर चलाउनुहुन्छ (जस्तै, ब्याडमिन्टन, टेबल टेनिस, क्रिकेटमा बलिङ गर्ने, आदि)	१	२	३	४	५
२०. एक ठाउँबाट अर्को ठाउँमा गाडीमा जान	१	२	३	४	५
२१. यौन क्रियाकलापहरु	१	२	३	४	५

पाखुरा, काँध र हातको असक्षमताहरु (DASH-NP)

	कतिपनि छैन	थोरै	मध्यम	धेरै	अत्याधिक
२२. गत हप्तामा, तपाईको पाखुरा, हात अथवा काँधको समस्याले आफ्नो परिवार, साथीहरु, छिमेकिहरु अथवा समुहहरुसँग गरिने सामान्य सामाजिक क्रियाकलापहरुमा कति हदसम्म बाँधा पुर्यायो ? (अंकमा गोलो लगाउनुस्)	१	२	३	४	५

	सिमित थिएन	थोरै सिमित	मध्यम सिमित	धेरै सिमित	गर्नै सकिनँ
२३. गत हप्तामा, तपाईको पाखुरा, हात अथवा काँधको समस्याको कारणले, तपाईले काममा अथवा अन्य दैनिक क्रियाकलापहरुमा कति सिमित हुनुभयो?	१	२	३	४	५

गत हप्ता भएको निम्न समस्याहरुको मात्राको कृपया मूल्याङ्कन गर्नहोस् । (अंकमा गोलो लगाउनुस्)

	छैन	थोरै	मध्यम	धेरै	अत्याधिक
२४. पाखुरा, काँध अथवा हातको दुखाई	१	२	३	४	५
२५. कुनै बिशेष क्रियाकलाप गर्दा पाखुरा, काँध अथवा हातमा हुने दुखाई	१	२	३	४	५
२६. पाखुरा, काँध अथवा हातमा झम-झम हुने	१	२	३	४	५
२७. पाखुरा, काँध अथवा हातको कमजोरी	१	२	३	४	५
२८. पाखुरा, काँध अथवा हात जाम हुने	१	२	३	४	५

	गाह्रो छैन	थोरै गाह्रो	मध्यम गाह्रो	धेरै गाह्रो	यति धेरै गाह्रो कि म सुत्न सक्दिन
२९. गएको हप्तामा, तपाईको पाखुरा, काँध अथवा हातको दुखाईको कारणले सुत्न कत्तिको गाह्रो भएको छ ? (अंकमा गोलो लगाउनुस्)	१	२	३	४	५

	एकदमै असहमत	असहमत	न त सहमत न त असहमत	सहमत	एकदमै सहमत
३०. मेरो पाखुरा, काँध अथवा हातको समस्याको कारणले आफुलाई कम सक्षम, कम आत्मनिर्भर अथवा कम उपयोगी महसुश गर्छु । (अंकमा गोलो लगाउनुस्)	१	२	३	४	५

पाखुरा, काँध र हातको असक्षमताहरु (DASH-NP)

कार्य भाग (वैकल्पिक)

निम्न प्रश्नहरुले तपाईको पाखुरा, हात अथवा काँधको समस्याको कारणले तपाईको कार्यक्षमतामा पारेको असरको बारे सोध्छ (घरको काम यदि यो तपाईको प्रमुख काम हो भने) ।

कृपया, तपाईको काम उल्लेख गर्नुहोस्: ____________________

☐ म काम गर्दिन ।(तपाईले यो भाग छोड्न सक्नुहुन्छ ।)

गत हप्ताको तपाईको शारीरिक क्षमताको राम्रो बयान गर्न कृपया, एक संख्यामा गोलो लगाउनुहोस्। तपाईलाई कत्ति गाह्रो थियो ?

	गाह्रो थिएन	थोरै गाह्रो	मध्यम गाह्रो	धेरै गाह्रो	गर्न सकिँन
१. सामान्य तरिकाले आफ्नो काम गर्न ?	१	२	३	४	५
२. तपाईको पाखुरा, काँध अथवा हातको दुखाईको कारणले सधैको काम गर्न ?	१	२	३	४	५
३. आफुले चाहे जस्तै राम्रोसंग काम गर्न ?	१	२	३	४	५
४. सधै जस्तो समय खर्च गरेर आफ्नो काम गर्न ?	१	२	३	४	५

खेलकुद/कला प्रदर्शन भाग (वैकल्पिक)

निम्न प्रश्नहरु तपाईको पाखुरा, हात अथवा काँधको समस्याको कारणले तपाईको खेलमा वा सांगीतिक बाजा बजाउनमा पारेको असरको बारेमा सम्बन्धित छ यदि एक भन्दा बढि खेल खेल्नुहुन्छ वा सांगीतिक बाजा बजाउनुहुन्छ (वा दुवै) भने ति मध्य सबैभन्दा महत्त्वपूर्ण कृयाकलापको आधारमा कृपया उत्तर दिनुहोस् ।

कृपया, तपाईको सबैभन्दा महत्वपूर्ण खेल वा बाजा उल्लेख गर्नुहोस्: ____________________

☐ म खेल खेल्दिन वा कुनै बाजा बजाउदिन।(तपाईले यो भाग छोड्न सक्नुहुन्छ ।)

गत हप्ताको तपाईको शारीरिक क्षमताको राम्रो बयान गर्न कृपया, एक संख्यामा गोलो लगाउनुहोस्। तपाईलाई केहि गाह्रो थियो ?

	गाह्रो थिएन	थोरै गाह्रो	मध्यम गाह्रो	धेरै गाह्रो	गर्न सकिँन
१. आफ्नो तरिकाले खेल खेल्न वा बाजा बजाउँन?	१	२	३	४	५
२. तपाईको पाखुरा, काँध अथवा हातको दुखाईको कारणले सधैको खेल खेल्न वा बाजा बजाउँन?	१	२	३	४	५
३. आफुले चाहे जस्तै सधैको खेल खेल्न वा बाजा बजाउँन?	१	२	३	४	५
४. सधै जस्तो समय खर्च गरेर आफ्नो खेल खेल्न वा बाजा बजाउँन?	१	२	३	४	५
